# Bi-Allelic Loss-of-Function Variant in MAN1B1 Cause Rafiq Syndrome and Developmental Delay

**DOI:** 10.3390/ijms26167820

**Published:** 2025-08-14

**Authors:** Liyu Zang, Yaoling Han, Qiumeng Zhang, Si Luo, Zhengmao Hu, Kun Xia, Ashfaque Ahmed, Qi Tian

**Affiliations:** 1 MOE Key Laboratory of Rare Pediatric Diseases & Hunan Key Laboratory of Medical Genetics of the School of Life Sciences, Central South University, Changsha 410078, China; zangliyu@sklmg.edu.cn (L.Z.); hanyaoling@sklmg.edu.cn (Y.H.); zhangqiumeng@sklmg.edu.cn (Q.Z.); luosi@sklmg.edu.cn (S.L.); huzhengmao@sklmg.edu.cn (Z.H.); xiakun@sklmg.edu.cn (K.X.); 2 MOE Key Laboratory of Rare Pediatric Diseases, School of Basic Medicine, Hengyang Medical College, University of South China, Hengyang 421001, China; 3 Furong Laboratory, Changsha 410078, China

**Keywords:** *MAN1B1*, Rafiq syndrome, intellectual disability, neuronal development

## Abstract

Rafiq syndrome (RAFQS) is a rare autosomal recessive disorder that is classified as a type II congenital disorder of glycosylation (CDG-II), and caused by *MAN1B1* gene mutation. To date, 24 pathogenic *MAN1B1* mutations have been reported in association with MAN1B1-CDG. However, the underlying pathogenic mechanisms remain poorly understood. In this study, we recruited a consanguineous family from Pakistan with multiple affected individuals exhibiting mild facial dysmorphism, developmental delay, and intellectual disability. Utilizing exome sequencing and homozygosity mapping, we identified a novel *MAN1B1* mutation (c.772_775del) that co-segregated with RAFQS in this family. Analysis of public single-cell transcriptomic data revealed that *MAN1B1* is predominantly expressed in dorsal progenitors and intermediate excitatory neurons during human brain development. Knockdown of *Man1b1* in primarily cultured mouse excitatory neurons disrupted axon growth, dendrite formation, and spine maturation, and could not be rescued by truncated variants identified in the family. Furthermore, in utero, electroporation experiments revealed that *Man1b1* knockdown in the murine cortex impaired neural stem cells’ proliferation and differentiation, as well as cortical neuron migration. Collectively, these findings elucidate a critical role for *MAN1B1* in the etiology of RAFQS and demonstrate that loss-of-function mutation in *MAN1B1* disrupt neuro-developmental processes, providing mechanistic insights into the pathogenesis of this disorder.

## 1. Introduction

Congenital disorders of glycosylation (CDG) are inherited metabolic disorders caused by defects in the synthesis and processing of the asparagine (ASN)-linked oligosaccharides of glycoproteins [1,2,3]. CDG are broadly classified into two categories: CDG-I (involving defects in the biosynthesis, assembly, and transfer of endoplasmic reticulum (ER)-localized dolichol-linked oligosaccharide Glc_3_Man_9_GlcNAc_2_ to proteins) and CDG-II (resulting from impaired remodeling of protein-bound oligosaccharides in the Golgi apparatus) [4,5]. Notably, Rafiq syndrome (RAFQS), an autosomal recessive CDG-II subtype, is caused by bi-allelic mutations in the *MAN1B1* gene. To date, 46 confirmed RAFQS cases across 35 unrelated families have been documented [6,7]. Affected individuals predominantly manifest mild to moderate intellectual disability (ID), frequently accompanied by motor developmental delay, truncal obesity, and muscular myopathies. Notably, distinctive craniofacial dysmorphism is consistently observed, characterized by a broad nasal bridge with hypertelorism, laterally thinned eyebrows, a prominent nose with a bulbous tip, and a thin upper vermilion border. Moreover, episodes of aggression and epilepsy have been documented in a subset of patients [8,9]. In contrast to Phospho-mannomutase 2 congenital disorders of glycosylation (PMM2-CDG) [10], MAN1B1-CDG lacks systemic involvement and manifests with earlier disease onset, posing greater diagnostic challenges clinically. Due to the disorder’s low prevalence, clinical heterogeneity, and absence of pathognomonic features, diagnosis remains challenging. Consequently, diagnostic strategies increasingly rely on whole-exome sequencing or Electrospray Ionization (ESI) mass spectrometry to detect glycosylation abnormalities [11].

The *MAN1B1* gene, mapped to chromosomal locus 9q34.3, encodes endoplasmic reticulum mannosyl-oligosaccharide 1,2-α-mannosidase (ERManI), a type II transmembrane protein. As a member of glycosyl hydrolase family 47, ERManI localizes to the Golgi apparatus and participates in N-glycosylation within the secretory pathway [12] which constitutes an essential post-translational modification for glycoprotein maturation in eukaryotes. Moreover, MAN1B1 functions as a critical regulator in the endoplasmic reticulum-associated degradation (ERAD) pathway, functioning as a quality control checkpoint by precisely excising terminal α1,2-mannose residues from misfolded glycoproteins escaping the ER [13].

Although 24 pathogenic *MAN1B1* mutations have been associated with RAFQS, the molecular mechanisms underlying disease pathogenesis remain elusive. To elucidate the neurobiological consequences of *MAN1B1* deficiency, this study employed exome sequencing coupled with homozygosity mapping, single-cell RNA sequencing analysis, in utero electroporation, and in vitro neuronal cultures. Specifically, we systematically examined the role of MAN1B1 in cortical neurogenesis and neuronal morphogenesis, thereby providing mechanistic insights into the etiology of intellectual disability in RAFQS.

## 2. Results

### 2.1. Clinical Evaluation of Affected Individuals

**Figure 1:**
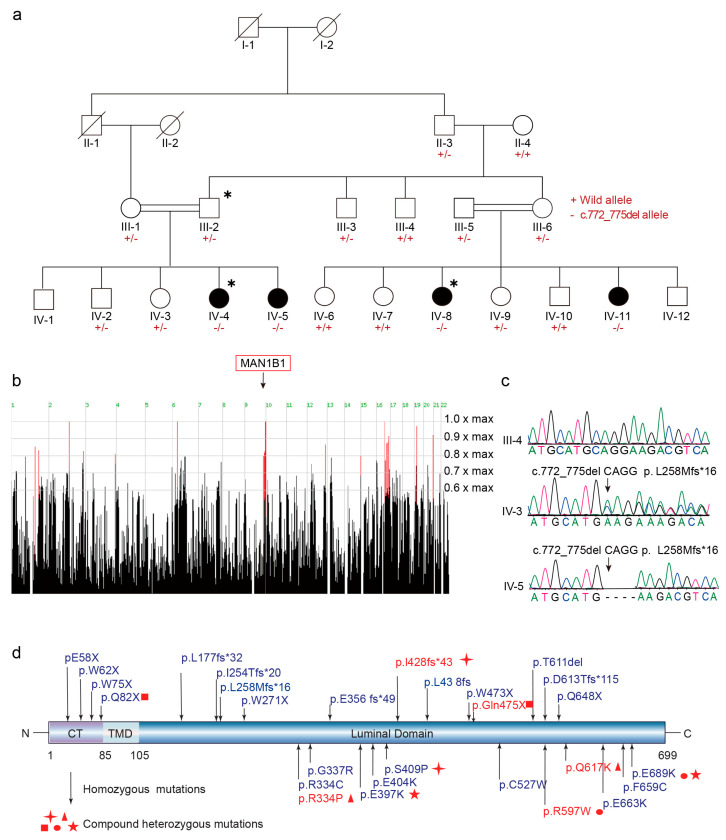
Genetic studies of the intellectual disability family. (**a**) Pedigree of the Pakistani consanguineous family in this study, and Sanger sequencing chromatograms of the variation. ∗, Samples whose DNA have been selected for ES; +, stands for wild-type allele; −, stands for alternative allele. (**b**) Screen shots show the ID family’s genome-wide homozygosity scores produced by HomozygosityMapper. Red bars indicated the most promising genomic regions. (**c**) Sanger sequence of c.772_775del CAGG, p. L258Mfs*16 variant in MAN1B1 gene. (**d**) MAN1B1 mutations reported to be associated with intellectual disability. ╋▲■●★, identical symbols denote compound heterozygous combinations within a single patient.

A four-generation consanguineous family originating from a rural region in Southern Pakistan was recruited for this study (Figure 1a). The primary clinical phenotype observed in this family was intellectual disability (ID), with four affected female individuals and 20 unaffected members identified. All affected individuals exhibited mild to moderate intellectual disability and developmental delays, ranging in age from 2 to 16 years.

Notably, patients IV-4 and IV-5 (Supplementary Figure S1a,b) are siblings born to first-degree consanguineous parents, while patients IV-8 and IV-11 (Supplementary Figure S1c,d) are siblings whose parents share the same surname and self-reported consanguinity. All four patients were diagnosed with mild to moderate intellectual disability between 2 and 3 years of age. Consistent clinical features included impaired communication skills, social awkwardness, and significant learning difficulties. Specifically, the patients displayed poor attention spans, cognitive deficits, and an inability to comprehend monetary concepts. Although they required parental assistance for dressing, all were capable of independent bathroom use. Delayed speech development was universally observed.

Patient IV-4 presents with mild facial asymmetry, strabismus, and thin upper lip. She manifests marked hyperactivity, emotional lability, linguistic impairment, social withdrawal (particularly toward unfamiliar individuals), attentional deficits, and learning disabilities. Patient IV-5 manifests marked hyperactivity, emotional lability, linguistic impairment, social withdrawal (particularly toward unfamiliar individuals), attentional deficits, and learning disabilities, though capable of basic social engagement and ambulation within familiar village environments. Patient IV-8 exhibits mild craniofacial dysmorphism and demonstrates capacity for rudimentary social interaction but displays affective instability and intermittent aggressive outbursts. Notably, Patients IV-4, IV-5, and IV-8 retain topographic memory of local pathways. Case IV-11 manifests strabismus and prominent nose with bulbous tip. She presents with impaired self-care capacity (including dressing apraxia), global expressive aphasia, marked xenophobia, and absence of self-preservation awareness. A comprehensive summary of the clinical characteristics is provided in Table 1.

### 2.2. MAN1B1 Is the Candidate Causal Gene in the Family

To identify the genetic basis of the observed phenotype, whole-exome sequencing (WES) was performed on two affected individuals (IV-4, IV-8) and one unaffected family member (III-2) in the family (Figure 1a). Homozygosity mapping revealed candidate homozygous loci on chromosomes 2, 6, 9, 16, and 19 (Figure 1b). By integrating homozygosity mapping with exome sequencing data and applying a stringent filtration strategy under the autosomal recessive inheritance model, a single rare deletion variant in *MAN1B1* (NM_016219.5; c.772_775del) was identified on chromosome 9q. Sanger sequencing confirmed that this variant co-segregated with intellectual disability (ID) in the family (Figure 1a,c). Bioinformatics analysis predicted that this deletion results in a frameshift mutation (p. L258Mfs*16), leading to a truncated protein lacking the majority of the catalytic domain, which is essential for enzymatic activity.

Notably, the aforementioned variant was absent in the Genome Aggregation Database (gnomAD), Exome Aggregation Consortium (ExAC), and 1000 Genomes Project databases. Consequently, in accordance with the American College of Medical Genetics and Genomics (ACMG) guidelines, this frameshift variant was classified as pathogenic, supported by the following evidence criteria: PVS1 (null variant in a gene where loss-of-function is an established disease mechanism), PM2 (absent in population databases; supporting strength), PP1 (co-segregation with disease), and PS3 (moderate-strength functional evidence).

### 2.3. Expression of MAN1B1 Is Positively Correlated with the Development of Excitatory Neurons in the Human Brain

**Figure 2:**
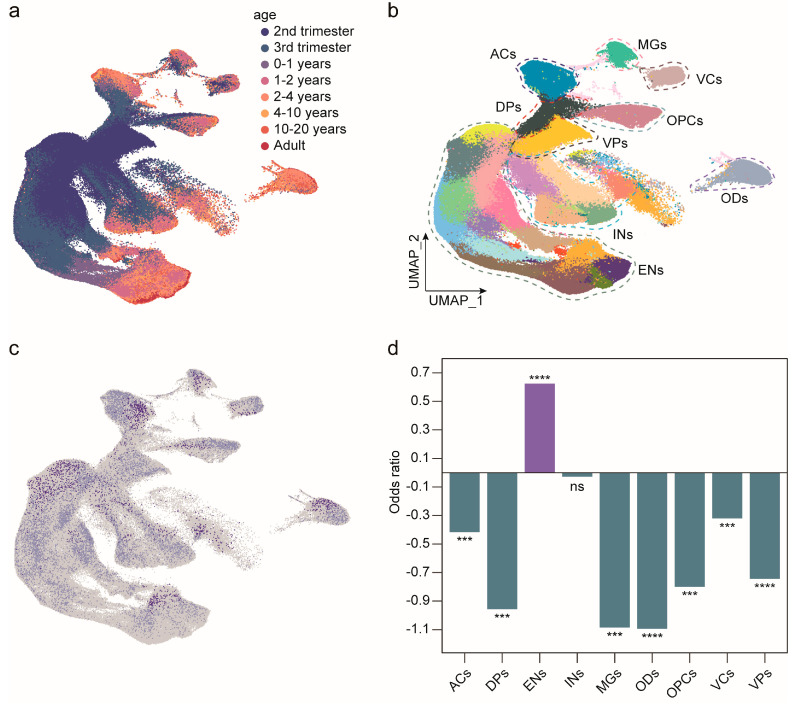
Temporal-spatial expression pattern of *MAN1B1* in the developing human brain. (**a**) Nuclei in whole data labeled by their developmental stage. Cluster numbers and biological interpretations are available from the source study. (**b**) Major clustering of public datasets labeled with main cell lines. (**c**) Distribution of *MAN1B1* expression in all cells associated with (**a**,**b**). (**d**) Relative enrichment of *MAN1B1* expression in different cell types. *p* values calculated by Wilcoxon rank sum test and adjusted by Bonferroni method (n = 9). ACs, astrocytes; DPs, dorsal progenitors; ENs, excitatory neurons; INs, interneurons; MGs, microglia; ODs, oligodendrocytes; OPCs, oligodendrocyte progenitor cells; VCs, vascular cells; VPs, ventral progenitors; ns, no significance, ***, *p* < 0.001, ****, *p* < 0.0001.

*MAN1B1* is widely expressed in the brain, but its specific expression pattern is unknown. To investigate the expression pattern of *MAN1B1* during cortical development, we analyzed single-cell RNA sequencing data from the human cerebral cortex using the public resources (UCSC cell browser). We broadly categorized them into nine cell types: astrocytes, dorsal progenitors, excitatory neurons, interneurons, microglia, oligodendrocytes, oligodendrocyte progenitor cells, vascular cells, and ventral progenitors (Figure 2a,b). This result showed that *MAN1B1* was expressed in all subclasses (Figure 2c) and was increasingly expressed in excitatory neurons with aging (Figure 2d). The expression pattern suggests that *MAN1B1* plays an important role in the development of cortical neurons.

Then we explored the temporal expression profile of *Man1b1* in the mouse cerebral cortex. As shown in Supplementary Figure S2a, *Man1b1* is expressed during the embryonic period and gradually increases after birth, mimicking the expression pattern in the human cortical cortex. Since the MAN1B1 amino acid sequences of the human and mouse are similar (78.35% identified in amino acid sequence) and functionally conserved (Supplementary Figure S2c), the mouse model is suitable to study MAN1B1 function during cerebral cortex development.

### 2.4. Man1b1 Deficiency Impairs Axonal Outgrowth and Synapse Development in the Mouse Brain

To elucidate the effects of MAN1B1 deficiency during neuron genesis, we constructed four Man1b1 shRNAs to knock down Man1b1 expression in primarily cultured mouse pyramidal cortical neurons. As shown in Supplementary Figure S2b, the most effective Msh3 was chosen for downstream analysis.

**Figure 3:**
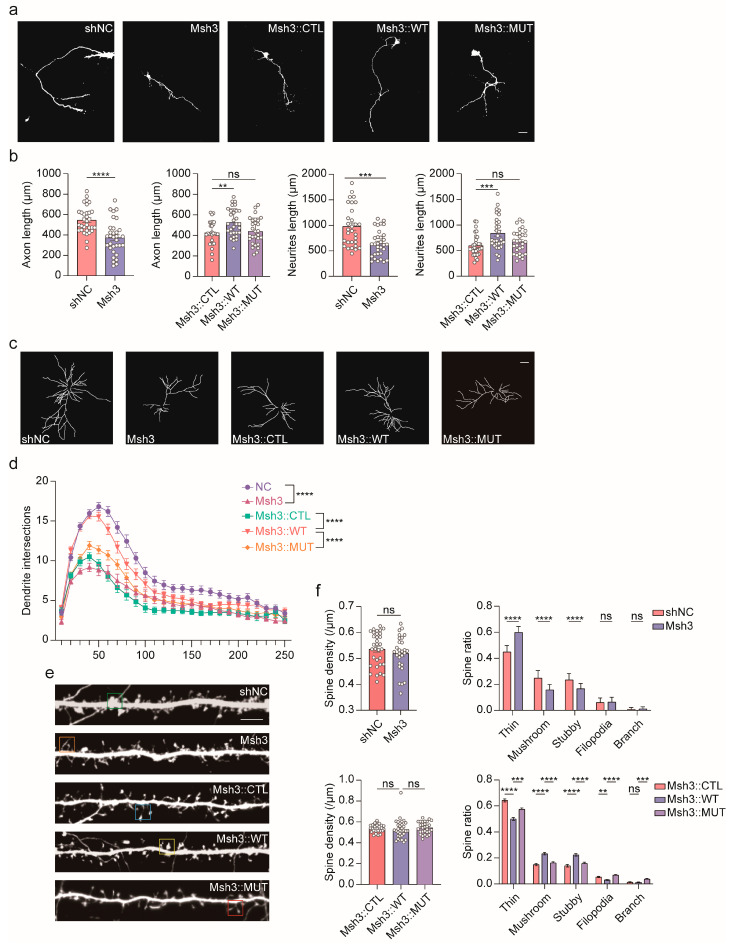
Disruption of MAN1B1 impairs axonal outgrowth and synapse development. (**a**,**b**) Man1b1 KD inhibits neurite and axon growth (each n = 30). Neurons were stained with DAPI (nucleus) and green fluorescent protein (neuronal morphology). Scale bar, 50 μm. Statistical significance was determined by one-way ANOVA. (**c**,**d**) Man1b1 KD inhibits dendritic complexity (each n = 30). Scale bar, 50 μm. Statistical significance was determined by two-way ANOVA. (**e**,**f**) Man1b1 KD disrupts dendritic spine morphogenesis and maturation (30 cells from two independent cultures using two mice for each condition). Scale bar, 10 μm. Statistical significance was determined by one-way ANOVA. All quantification data are shown as the mean ± SEM. ns, no significance, *p* > 0.05; **, *p* < 0.001; ***, *p* < 0.0001; ****, *p* < 0.00001. Green square, stubby; Orange square, filopodia; Blue square, thin; Yellow square, mushroom; Red square, branch.

The pyramidal cortical neurons were primarily cultured on day DIV1, and Msh3 was introduced into the cells on day DIV2. The cells were harvested on day DIV5, and the axon and total neurite length were analyzed. As shown in Figure 3a,b, *Man1b1* knockdown resulted in shorter axon and total neurite lengths, and human MAN1B1^WT^ can rescue these differences, but MAN1B1^MUT^ failed. We also observed a significant reduction in dendritic complexity after knockdown *Man1b1* expression (Figure 3c,d). This reduction can be rescued by MAN1B1^WT^ but not by MAN1B1^MUT^. Thus, we postulate that MAN1B1 might participate in the neuronal maturation process, particularly in determining dendrite complexity and neurite extrusion. MAN1B1^MUT^ is a loss of function mutation.

Next, we assessed the spine genesis and maturation in *Man1b1* knockdown primarily cultured pyramidal cortical neurons. The neurons were transfected on day DIV6, and the spines were analyzed on day DIV18. As shown in Figure 3e,f, the dendritic spine density was unaffected by knocking down *Man1b1*, but the number of matured spines (mushrooms and short stubby spines) was reduced, whereas the number of immature spines (thin spines) was significantly increased. The overexpression of MAN1B1^WT^ can rescue these phenotypes, whereas MAN1B1^MUT^ cannot. These results suggest that MAN1B1 plays a critical role in spine maturation and demonstrate that MAN1B1^MUT^ is a loss-of-function mutation.

### 2.5. Man1b1 Deficiency Promotes Mouse Neural Stem Cells’ Proliferation and Inhibits Migration

**Figure 4:**
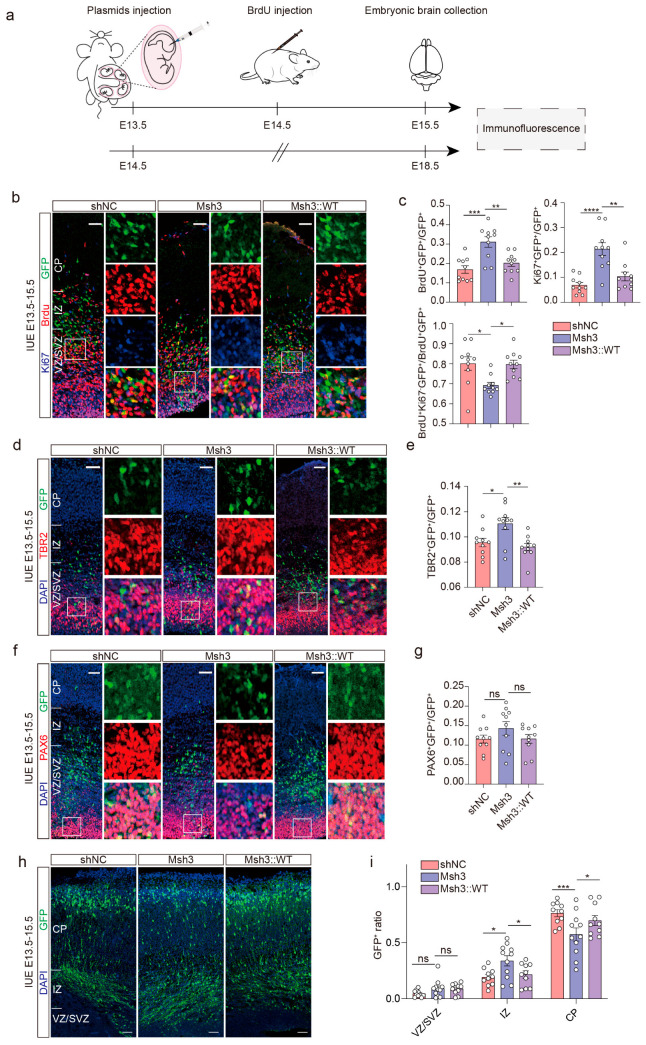
MAN1B1 disruption in the developing brain disturbs neurogenesis. (**a**) Schematic diagram of in utero electroporation. (**b**,**c**) Comparison of Ki67+ (red) population, GFP+ (green) population, and GFP+ proportion of apical surface from knockdown of Man1b1 in E15.5 mouse cortices electroporated at E13.5 (each n = 5). The square denotes the region magnified in the right panel of the figure. (**d**,**e**) Increased number of TBR2+ (red) differentiated IPs caused by Man1b1 knockdown in E15.5 mouse cortices electroporated at E13.5 (each n = 5). (**f**,**g**) No significant number of PAX6+ (red) NSCs changed caused by Man1b1 knockdown in E15.5 mouse cortices electroporated at E13.5 (each n = 5). (**h**,**i**) Impairment of neuronal migration by shRNA-mediated knockdown of Man1b1 in E18.5 mouse cortices electroporated at E14.5 (each n = 5). CP, cortical plate, the outer layer of the developing cerebral cortex where immature neurons arrive at the corresponding layer from the inside out according to a strict time sequence; IZ, intermediate zone, a transient region between the ventricular zone and the cortical plate during brain development; VZ, ventricular; SVZ, subventricular zone. SVZ is a thin strip-like structure surrounding the lateral wall of the lateral ventricle below the corpus callosum, containing neural progenitor cells. Square denotes co-labeled cells. Scale bars, 50 μm. All statistical significance were determined by one-way ANOVA. All data are represented as mean ± SEM. ns, no significance, *p* > 0.05; *, *p* < 0.05; **, *p* < 0.01; ***, *p* < 0.001; ****, *p* < 0.0001.

To investigate the role of MAN1B1 in the development of the mouse cerebral cortex in vivo, we utilized Msh3 to knockdown the expression of *Man1b1* in the mouse cerebral cortex. The Msh3, MAN1B1^WT^, and MAN1B1^MUT^ plasmids were in utero electroporated into the lateral ventricle zone of E13.5 mice, and the BrdU was intraperitoneally injected into the pregnant mouse 24 h later (Figure 4a). The BrdU was used to label cells that experienced the S-phase of the cell cycle within a 24-hour period, and the Ki67 was employed to label all cells except those in the G0 phase at the specific point in time. Consequently, the combination of BrdU^+^ and Ki67^−^ allows for the identification of cells that have undergone proliferation and exited the cell cycle [14]. On day E15.5, cell proliferation was analyzed by immuno-histo-fluorescence staining. In the case of *Man1b1* knockdown, the proportion of BrdU^+^GFP^+^/GFP^+^ and Ki67^+^GFP^+^/GFP^+^ was significantly increased compared with the shNC group, showing enhanced proliferation activity. The Ki67^-^BrdU^+^GFP^+^/BrdU^+^GFP^+^ ratio was significantly decreased in the Msh3 group, indicating that knockdown of *Man1b1* decreased the number of cells exiting the cell cycle after mitosis within 24 h (Figure 4b,c). At the same time, overexpression of MAN1B1^WT^ was able to rescue the proliferation differences. These results suggest that the knockdown of *Man1b1* may promote the proliferative process of neural progenitor cells.

Next, we assessed the proliferation activity of radial glial [15] (PAX6 positive) and intermediate progenitor (TBR2 positive) [16] cells after knocking down *Man1b1*. The ratio of TBR2^+^GFP^+^/GFP^+^ was significantly higher in the Msh3 group compared with shNC, indicating an increase in the number of intermediate progenitor cells 48 h after *Man1b1* interference (Figure 4d,e); the ratio of Pax6^+^ GFP^+^/GFP^+^ did not change significantly compared with shNC, indicating that the proliferation rate of radial glial progenitor cells was intact (Figure 4f,g). These results suggest that MAN1B1 does not affect the level of radial glial cells but may regulate the balance of proliferation and differentiation in intermediate progenitor cells.

To investigate the effect on neuronal migration after knocking down the *Man1b1* expression, we performed IUE on E15.5 and harvest on E18.5. Following immunohistochemical staining, a significant reduction in GFP-labeled neurons migrating to the cortical plate (CP) was observed upon MAN1B1 knockdown. Correspondingly, the proportion of neurons retained within the intermediate zone (IZ) of lower cortical layers was markedly increased. Conversely, no significant alterations were detected in neuronal distribution within the ventricular zone (VZ) or subventricular zone (SVZ) (Figure 4h,i). MAN1B1 deficiency may inhibit neuron migration during cortical neurogenesis.

In summary, MAN1B1 deficiency inhibits mouse neural stem cells from exiting the cell cycle and impairs newborn neuron migration.

### 2.6. The MAN1B1 Mutation Causes Slower Degradation of Misfolded Proteins and Does Not Impact Endoplasmic Reticulum (ER) Stress

Emerging evidence has elucidated that MAN1B1 participates in the retrieval of endoplasmic reticulum-associated degradation (ERAD) substrates from the Golgi apparatus [17]. Notably, the cytoplasmic tail of MAN1B1 has been demonstrated to directly interact with coat protein complex I (COPI) components, thereby positioning this enzyme as a critical mediator in the pre-ERAD retrieval pathway for misfolded glycoproteins [12].

To evaluate MAN1B1’s role in misfolded protein degradation, we co-overexpressed wild-type or mutant *MAN1B1* plasmids with its substrates, two naturally occurring misfolded N-glycosylated variants of human alpha1antitrypsin (AAT), Null Hong Kong and Z (NHK or ATZ [18]), into CRISPR/Cas9-generated MAN1B1 knockout 293T cells.

**Figure 5:**
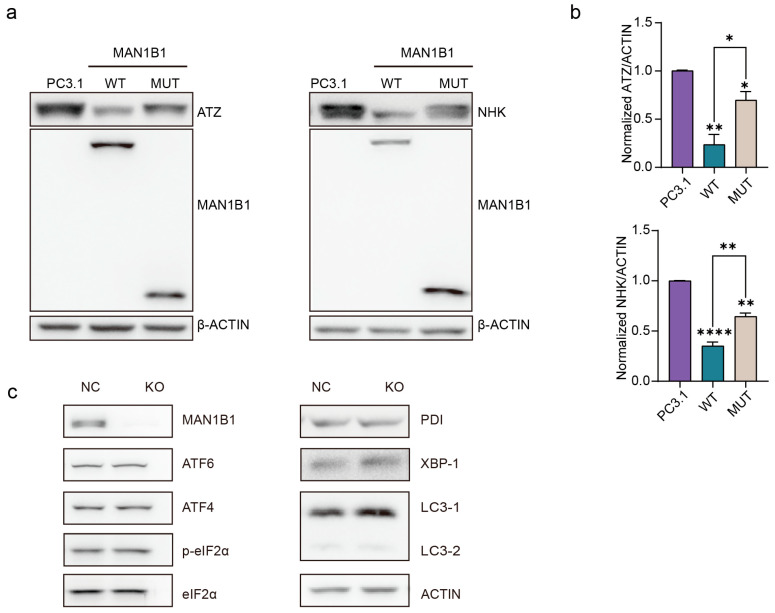
The MAN1B1 mutation causes slower degradation of misfolded proteins. (**a**,**b**) In the MAN1B1-KO cell line, the misfolded proteins NHK and ATZ were co-transfected with pcDNA3.1, WT, and MUT plasmids, respectively, for a period of 48 h. The protein lysates were then collected, and the results of the Western Blot assay were presented. Repeat each experiment 3 times. Statistical significance was determined by one-way ANOVA. (**c**) Western blotting indicated the proteins in extracts of HEK293T and MAN1B1-KO HEK293T cells. *, *p* < 0.05; **, *p* < 0.01; ****, *p* < 0.0001.

As shown in Figure 5a,b, wild-type MAN1B1 overexpression markedly enhanced substrate clearance, achieving approximately 77% (0.7671 ± 0.1055) ATZ and 65% (0.6481 ± 0.04004) NHK degradation. In contrast, the MAN1B1 mutant exhibited significantly impaired activity, reducing ATZ and NHK by only ~30% (0.3049 ± 0.09436) and ~35% (0.3552 ± 0.04004), respectively. These results demonstrate that the MAN1B1 mutation substantially impairs the cellular proteostasis machinery’s ability to clear misfolded substrates.

Notably, subsequent analysis of endoplasmic reticulum (ER) stress markers showed no significant expression changes across experimental conditions (Figure 5c). Together, these findings indicate that while the MAN1B1 mutation compromises misfolded protein degradation capacity, this functional defect does not trigger compensatory ER stress responses.

## 3. Discussion

Almost all of the patients who have been reported to have mutations in MAN1B1 exhibit intellectual disability, whereas the relationship between MAN1B and neurological has never been reported. In this study, we identified a homozygous loss-of-function variant in MAN1B1 within a consanguineous family exhibiting intellectual disability (ID), thereby diagnosing the condition as Rafiq syndrome (RAFQS) through comprehensive genetic analysis. We delineated the clinical phenotypes of affected individuals, thereby expanding the spectrum of pathogenic *MAN1B1* mutations. Furthermore, our functional studies in mice revealed that MAN1B1 deficiency disrupts cortical neurogenesis. The presence of the Man1b1 deletion may promote the proliferation of neural progenitor cells and inhibit their differentiation, while also promoting the proliferation of intermediate progenitor cells. This study provided mechanistic insights into its contribution to neurodevelopmental disorders.

*MAN1B1* mutations were first linked to intellectual disability in 2011 [19] with three distinct mutations identified across five consanguineous families. Affected individuals exhibited developmental delays, cognitive impairments, and, in some cases, epilepsy, aggressive behavior, obesity, and facial dysmorphisms. Subsequent studies by Daisy Rymen et al. described additional clinical features, including skin laxity, joint hypermobility, and autism spectrum disorder [20]. One patient presents with macrocephaly, and another showed cerebellar hypoplasia with vermian atrophy. Subsequently, more and more patients with defective MAN1B1 were reported, showing individual variability in clinical phenotype, but most exhibited truncal obesity, joint hypermobility, a thin upper lip, short philtrum, and broad eyebrows [9]. According to the latest report, facial deformities were observed in approximately 84% of cases, whereas epilepsy and truncal obesity were documented in 16% and 56% of patients, respectively [7]. The characteristic facial phenotype notably includes strabismus, thin upper lips, prominent nose with bulbous tip, and short philtrum. Notably, none of the patients in the family presented with symptoms of obesity. This may be associated with the patient’s living environment and economic status. One patient also exhibited drooling, broadening the clinical phenotype of MAN1B1-CDG disease.

Additionally, by re-analyzing single-cell RNA sequencing data from the developing cerebral cortex, we demonstrated that *MAN1B1* is highly expressed in neuronal cell types, suggesting its critical role in neurodevelopment. In this report, we focused on the morphology of the cortical pyramidal neuronal dendrite to explore the abnormal function of the variants. We found that knockdown of *Man1b1* expression in cultured mouse pyramidal neurons resulted in shorter neuronal axons, fewer dendritic branches, and reduced dendritic spine maturation. Moreover, we found knockdown of *Man1b1* expression in embryonic mouse cortical cortex inhibited the intermediate progenitor cell cell-cycle exit and newborn neuron migration. These results suggest the crucial role of *MAN1B1* on the process of neuronal and cortical development. Although seminal studies have genetically linked bi-allelic *MAN1B1* mutations to intellectual disability (Rafiq syndrome) [7], the underlying neurobiological mechanisms driving this phenotype remain largely unresolved. Critically, our study provides the first direct functional evidence establishing that MAN1B1 serves an indispensable role in fundamental neurodevelopmental and neuronal maturation processes. Specifically, it is demonstrated that MAN1B1 deficiency impairs cortical neurogenesis by disrupting neural stem cell proliferation and differentiation, concurrently alters neuronal migration trajectories, and impedes neuronal morphogenesis via impaired axonal outgrowth, deficient dendritic arborization, and aberrant spine maturation.

The MAN1B1 protein has a glycoside hydrolase active region from amino acid 256 to 695 and is homologous to the mannosidase family. Previous researchers hypothesized that it was localized in the endoplasmic reticulum; however, subsequent studies have shown that MAN1B1 is localized in the Golgi apparatus and functions there to capture escaped misfolded proteins [13]. It was previously shown that MAN1B1 recruits COPI to transport misfolded proteins back into the endoplasmic reticulum. These misfolded proteins are refolded by endoplasmic reticulum chaperone proteins and associated lectins or eventually degraded by the proteasome via endoplasmic reticulum-associated degradation pathways [17]. In this article, we found no significant difference in protein expression level and subcellular localization in the mutant compared to the wild-type MAN1B1 (Supplementary Figure S3a,b). It has been found that MAN1B1 exists to promote degradation of misfolded AAT mutants in a manner unrelated to enzyme catalysis [12,21], which is also consistent with the fact that the mutant in this study still degraded 30% or 35% of the misfolded proteins (Figure 5a,b). Mutations that produce truncated proteins and loss of enzyme-active centers still partially degraded the misfolded proteins, which may be related to the presence of an unconventional mode of promoting degradation of the misfolded proteins in the N segment. However, the delayed degradation kinetics of the mutant protein (Supplementary Figure S3c,d) were observed, suggesting its aberrant accumulation within the Golgi compartment, which may disrupt Golgi homeostasis.

A limitation of this study is the absence of direct evidence for glycan processing defects in patient-derived neurons. To address this, future glycoproteomic profiling of induced pluripotent stem cell (iPSC)-differentiated neuronal models is warranted to validate the hypothesized mechanistic link between MAN1B1 related to aberrant glycosylation and neuronal defects.

## 4. Materials and Methods

A schematic of the experimental methodology is provided in Supplementary Figure S4.

### 4.1. Exome Sequencing, Bioinformatics Analysis, and Co-Segregation Validation

DNA was extracted from the venous blood of individuals in the four-generation consanguineous family. Exome sequencing was performed on two affected (IV-4, IV-8) and one unaffected subject (III-2). Briefly, genomic DNA was sheared and captured using the Agilent SureSelectXT Human All Exon V6 probe and sequencing on a HiSeq2500 (Illumina). Qualified reads were aligned to the reference human genome (hg38) using bwa 0.7.10. Variants were called with GATK 3.2.2 and annotated using ANNOVAR, version 201602 [22]. Homozygosity mapping was performed by an online tool, HomozygosityMapper (https://www.homozygositymapper.org/, accessed on 1 July 2023) [23], using exome sequencing data. Set the parameters as defaults, except limit the block length from 30 to 100 SNPs. Finally, based on recessive inheritance, screening out variants with allele frequency <0.01 in the gnomAD and 1000 Genomes Project databases, include non-synonymous variants in exon regions and splice-site variants. Finally, retain homozygous variants in IV-4 and IV-8 that are heterozygous in III-2.

The co-segregation analysis of candidate variants was performed by PCR and Sanger sequencing. The primers were designed using an online tool, Primer3 (https://primer3.ut.ee/, accessed on 1 July 2023), and the sequences are as follows: 5′-CCCAAACCCACCGTCATCTT-3′ (forward), 5′-TTGGGTGGCTGGACAAGAAC-3′ (reverse).

### 4.2. Expression Pattern of MAN1B1 in the Developing Human Brain

The single-cell RNA expression data were downloaded from the UCSC cell browser [24] (https://cells.ucsc.edu/?ds=human-cortical-dev, accessed on 1 July 2023), including the processed cell expression matrix, metadata, and the UMAP (Uniform Manifold Approximation and Projection) [25] coordinates. Cluster assignments for each cell were directly used from the source study. We used odds ratios (OR = (number of cells expressing *MAN1B1* in one cell type/number of cells in one cell type)/(number of cells expressing *MAN1B1* in other cell types/number of cells in other cell types)) to calculate the enrichment of *MAN1B1* for each cell type. The expression of *MAN1B1* over time was analyzed by monocle v3 [26,27]. Learn_graph and order cells functions are used to construct the trajectory graph.

### 4.3. Plasmid Construction

Full-length human *MAN1B1* (MAN1B1^WT^, NM_015323.5) cDNA was purchased from the American Type Culture Collection (Manassas, VA, USA). Then it was cloned into the pcDNA^TM^3.1/myc-His(−) B and pCAGGS-IRES-GFP vectors. MAN1B1^MUT^ was generated through amplification from the MAN1B1^WT^ expression construct using forward primer 5′-GAGGGCAGGAGAAGCGGA-3′ and reverse primer 5′-GGAAAATGCCACAGACCCAG-3′. Two naturally occurring misfolded N-glycosylated variants of human alpha1antitrypsin (AAT), Null Hong Kong (NHK), and Z (ATZ) cDNA construct were generated as previously described [21]. The shRNA plasmids were constructed using FUGW-H1 vector. The target sequences are as follows: Msh1, 5′-GCCTTGAGTTTATGGGTAGAA-3′; Msh2, 5′-GGAAATGGGTATCAGAGAACT-3′; Msh3, 5′-GCTGGAAGACTATGTTAAAGC-3′; and Msh4, 5′-GAGCCCTGAGATTGCACATTT-3′. The synonymous mutations were introduced into the *MAN1B1* expression vector to resist the shRNA.

### 4.4. Reverse Transcription and Real-Time PCR

Total RNAs from cultured cells were isolated using trizol reagent (15596026CN, Invitrogen, Waltham, MA, USA), and cDNA was synthesized using the RevertAid First Strand cDNA Synthesis Kit (K1622, Thermo Fisher Scientific, Waltham, MA, USA). Real-time PCR was performed with SYBR Premix Ex Taq (Bio-Rad, Hercules, CA, USA), and the forward (5′-CCCTACCTATGTGCATTGTTCTC-3′) and reverse (5′-TCCGTGGAACATGATTGTTG-3′) primers were used to quantify the expression level of *Man1b1*.

### 4.5. Mouse Primary Neuron Culture and Transfection

Primary neurons were obtained from embryonic day 16.5 (E16.5) mice. The detailed operation and reagents referred to the protocol recording by Gerard M J Beaudoin III [28]. Primary neurons were cultured in neurobasal neuron medium containing 2% B27 and 1% GlutaMAX. ransfection of mixed plasmids (shRNAs:eGFP-N1 = 2:0.5 for interference experiments or shRNA:MAN1B1^WT^:eGFP-N1 = 1:1:0.5 for rescue experiments) into primary neurons using lipofectamine 2000 reagent (11668027, Thermo Fisher Scientific, Waltham, MA, USA) on day 2 (DIV2). The cells were fixed on DIV5 to analyze axon length. Cortical pyramidal neurons were transfected using a Calcium Phosphate Cell Transfection Kit (40803ES70, Beyotime Biotechnology, Shanghai, China) on DIV5 and fixed at DIV14 and DIV18 for dendrite and spine formation analysis separately.

### 4.6. Immunofluorescence

The cultured neurons were washed twice using 1× PBS, then fixed by 4% paraformaldehyde containing 4% sucrose prepared in 1× PBS for 15 min at room temperature. After, permeation by 0.1% Triton X-100 for 10 min and blocked by 5% BSA in 1× PBS for 30 min. Neurons were sequentially incubated with primary antibody overnight at 4 °C, and second antibody at room temperature for 1h. The nuclei were stained using DAPI (Diamidino-2-phenylindole, C1002, Beyotime Biotechnology, Shanghai, China) at room temperature for 10 min. Images of the stained DIV5 cortical neurons were acquired using a Leica MD5000B microscope (Leica Camera, Wetzlar, Germany) using a 10× objective. Dendritic arborization and dendritic spines were captured by Leica TCS SP5 II confocal microscope with 20× or 63× objective lens. The images were then analyzed with ImageJ software, version 1.53t.

### 4.7. In Utero Electroporation

The embryonic 14.5 (E14.5) or E15.5 embryos of C57BL/6J mice were selected for in utero electroporation. Briefly, the pregnant mice were anesthetized by isoflurane. The uterine horns were exposed, and a 2 μg/μL plasmid mixed (shRNAs:eGFP-N1 = 2:0.5 for interference experiments or shRNA:MAN1B1^WT^:eGFP-N1 = 1:1:0.5 for rescue experiments) with Fast Green (0.1 mg/mL mixture) was injected into the lateral ventricle of the fetal brain. Then, electroporated by an Electro Square Porator machine (ECM830, Harvard Bioscience BTX, Holliston, MA, USA). The brain of each embryo was dissected and cryo-sectioned to 25 μm thickness 48 or 96 h post electroporation. Immunofluorescence staining was performed, and pictures were captured by confocal microscope (TCS SP5, LEICA, Wetzlar, Germany). The images were analyzed and illustrated using ImageJ software and Adobe Illustrator, version 24.2.3. To analyze the neuronal cell proliferation after interference, BrdU (B5002, Sigma, Burlington, MA, USA) was injected 24 h after in utero electroporation, and fetal mice were collected at 48 h. The primary antibodies used are as follows: mouse anti-BrdU (1:300, MAB3510, Millipore, Burlington, MA, USA), rabbit anti-PAX6 (1:400, ab195045, Abcam, Cambridge, UK), rabbit anti-TBR2 (1:400, ab23345, Abcam, Cambridge, UK), rabbit anti-Ki67 (1:400, D3B5, Cell Signaling Technology, Danvers, MA, USA), chicken anti-GFP (1:500, GFP-1020, Aves, Davis, CA, USA), and DAPI were used for staining nuclei. The second antibody used are as follows: anti-rabbit Cyanine3 (1:500, A10522, Thermo Fisher Scientific, Waltham, MA, USA), anti-mouse Cyanine5 (1:500, A10524 Thermo Fisher Scientific, Waltham, MA, USA), and anti-chicken Alexa Fluor™ 488 (1:500, A11039, Thermo Fisher Scientific, Waltham, MA, USA).

### 4.8. Transient Transfection and Western Blotting

MAN1B1 knockout 293T cells were cultured in a 6-well plate and 2 μg (MAN1B1^WT^ or MAN1B1^MUT^: NHK or ATZ = 1:2) were transfected according to the lipo2000 instructions. Approximately 48 h after transfection, 5 × 10^6^ cells were collected and cells were lysed with 1 mL SDS Lysis Buffer (P0013G, Beyotime Biotechnology, Shanghai, China). Twenty microgram proteins was separated by SDS/PAGE as previously described [18]. Anti-ACTIN was purchased from Sigma-Aldrich. Anti-MAN1B1 was purchased from thermo (PA5-104494, Thermo Fisher Scientific, Waltham, MA, USA). Anti-human AAT antibodies were purchased from MP Biomedicals (Solon, OH, USA). Endoplasmic reticulum stress-related markers were purchased from AiFang biological (Changsha, China).

### 4.9. Statistical Analysis

All functional assay data were repeated at least three times. Data were analyzed by GraphPad Prism version 9.3.1 using paired or unpaired Student’s *t*-tests, one-way ANOVA with Dunnett’s post-test, Bonferroni’s test, or two-way ANOVA with Bonferroni’s post-test.

## 5. Conclusions

In this study, we identified a novel pathogenic variant in *MAN1B1* (NM_016219.5; c.772_775del) in a consanguineous RAFQS family from Pakistan. Our findings demonstrate that MAN1B1 deficiency disrupts neuronal and cortical development, and that loss-of-function mutations in MAN1B1 contribute to intellectual disability through impaired neurogenesis.

## Figures and Tables

**Table 1:** Clinical characteristics in ID family.

Patient ID	IV-4	IV-5	IV-8	IV-11
Gender	female	female	female	female
Age at last evaluation	16 years	6 years	10 years	4 years
Head circumference (cm)	53.34	50.8	53.34	48.26
Height (cm)	152.4	96	121.92	80
Weight (kg)	35	24	25	15
BMI	15.1	26	16.8	23.4
Delayed walking (age)	+(2 years)	+(2 years)	+(2 years)	+(2 years)
Delayed speech (age)	+(3 years)	+(3 years)	+(3 years)	+(3 years)
Intellectual disability	Mild	Mild	Moderate	Moderate
Eye symptom	Strabismus	−	Strabismus	Strabismus
Dysarthria	+	+	+	+
Scoliosis	N/A	N/A	N/A	N/A
Lower limb weakness, feet equinovarus, or hammertoes	−	N/A	−	−

+, present; −, absent; N/A, not available.

## Data Availability

The authors confirm that all the data supporting the findings of this study are available in the article.

## Supplementary Materials

The following supporting information can be downloaded at: https://www.mdpi.com/article/10.3390/ijms26167820/s1.

## Abbreviations

The following abbreviations are used in this manuscript:

CDG-II	II congenital disorder of glycosylation
RAFQS	Rafiq syndrome
CDG	Congenital disorders of glycosylation
ERManI	endoplasmic reticulum mannosyl-oligosaccharide 1,2-α-mannosidase
ERAD	endoplasmic reticulum-associated degradation
E14.5	The embryonic 14.5
ID	intellectual disability
WES	whole-exome sequencing
gnomAD	Genome Aggregation Database
ExAC	Exome Aggregation Consortium
ACMG	American College of Medical Genetics and Genomics
DIV2	day 2
AAT	alpha1antitrypsin
NHK	Null Hong Kong
ER	endoplasmic reticulum
