# Supplementary material for: Bi-Allelic Loss-of-Function Variant in MAN1B1 Cause Rafiq Syndrome and Developmental Delay
Source: Int J Mol Sci. 2025 Aug 14;26(16):7820. doi: 10.3390/ijms26167820 (PMC12386520; doi:10.3390/ijms26167820)
Supplement: Supplementary file 1 [file ijms-26-07820-s001.zip › ijms-3748390-supplementary.pdf]

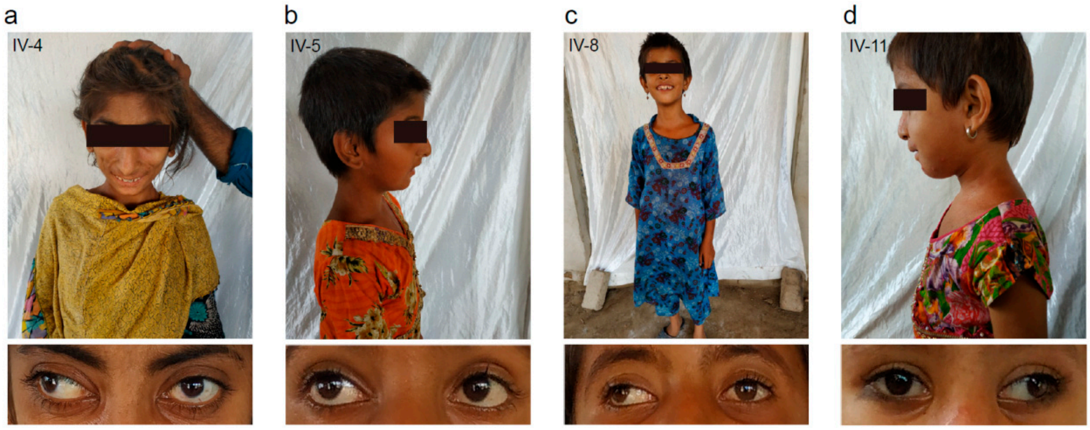

Supplementary Figure S1. (a) Clinical features of IV-4 at the age of 16 years. (b) Clinical features of IV-5 at the age of 6 years. (c) Clinical features of IV-8 at the age of 10 years. (d) Clinical features of IV-11 at the age of 4 years. Note the facial dysmorphism, strabismus (a,c,d), and thin upper lip.

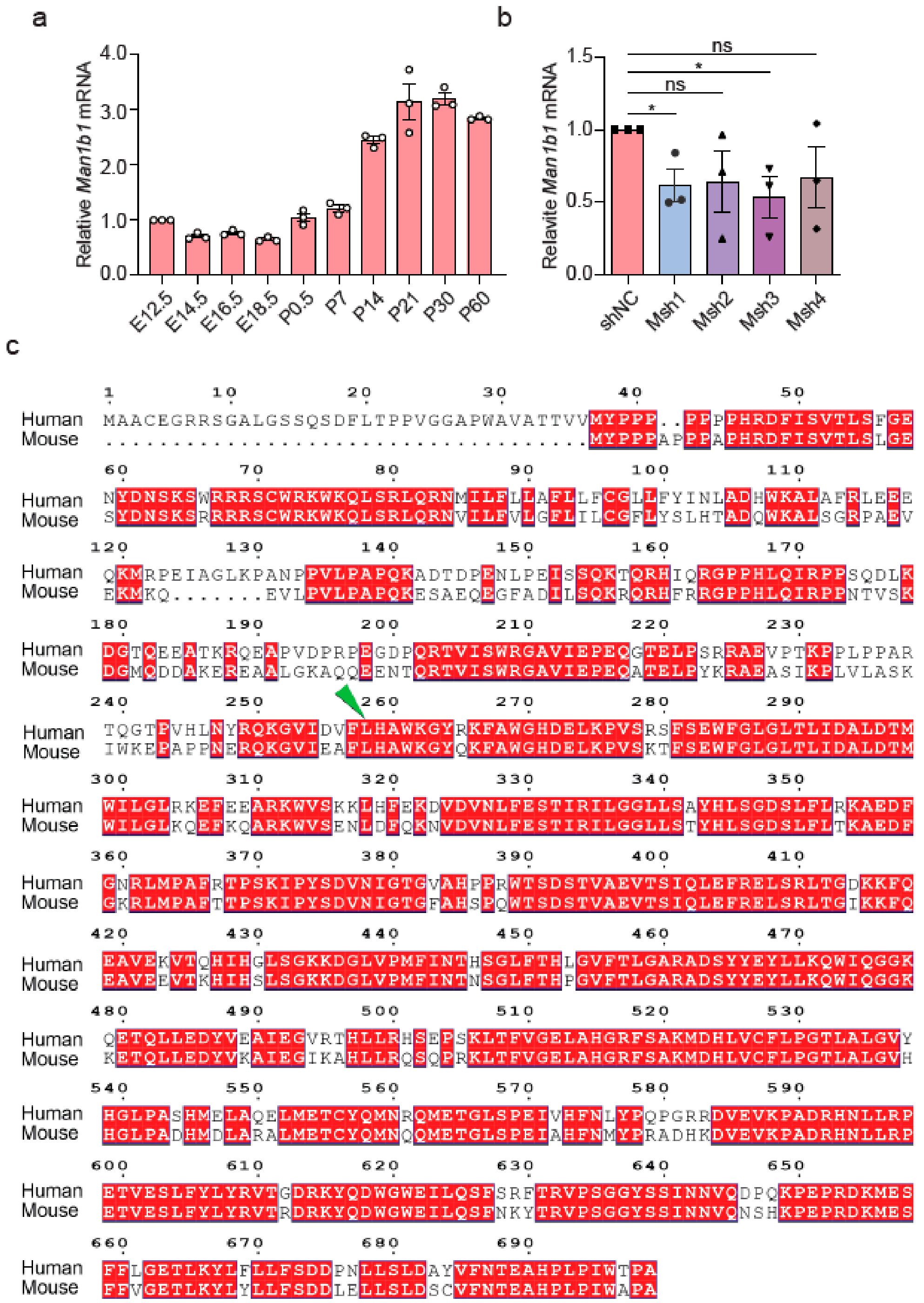

Supplementary Figure S2. (a) Temporal expression patterns of Man1b1 mRNA in the mouse brain (each n = 3). E, embryonic day; P, postnatal period. (b) Knocking down effect validation of 4 shRNAs targeting Man1b1 detected by RT-qPCR (each n = 3). Student's t-test. (c) Amino acids sequence alignment of human and mouse MAN1B1. Conserved residues between human MAN1B1 (NP_057303.2) and mouse Man1b1 (NP_001025154.1) are highlighted in red. Green arrows indicate mutation (c.772_775del) in our study's ID cases.

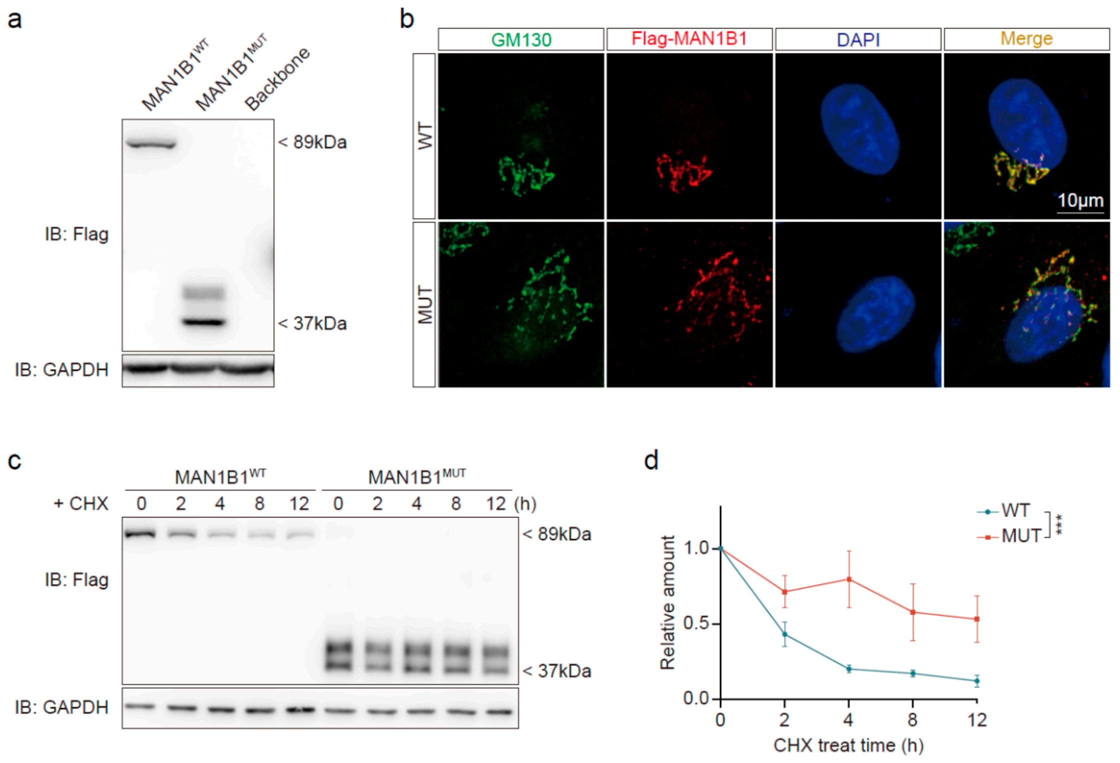

Supplementary Figure S3. (a) Immunoblots image of the exogenous expression of *MAN1B1* wild-type and mutant proteins. The mutation produced the p.L258Mfs*16 truncated protein. pCAGGS is empty vector. (b) Endogenous GOLGI marker protein GM130 was stained with secondary antibody Cyanine 5 (green), exogenous flag-tagged was stained with secondary antibody Cyanine3 (red), and nuclei were stained with 4.6-diamidino-2-phenylindole (blue). Scale bar = 5μm. (c) Immunoprecipitation plots of exogenous wild-type and mutant MAN1B1 overexpressed in HeLa cells for 12 hours after incubation in 50 μM cycloheximide. (d) Quantification of degradation rates of the wild-type and mutant from 0 to 12 h. All statistical significances were determined by paired Student's t-test. All data are represented as mean ± SD. * P < 0.05, ** P < 0.01, *** P < 0.001, **** P < 0.0001.

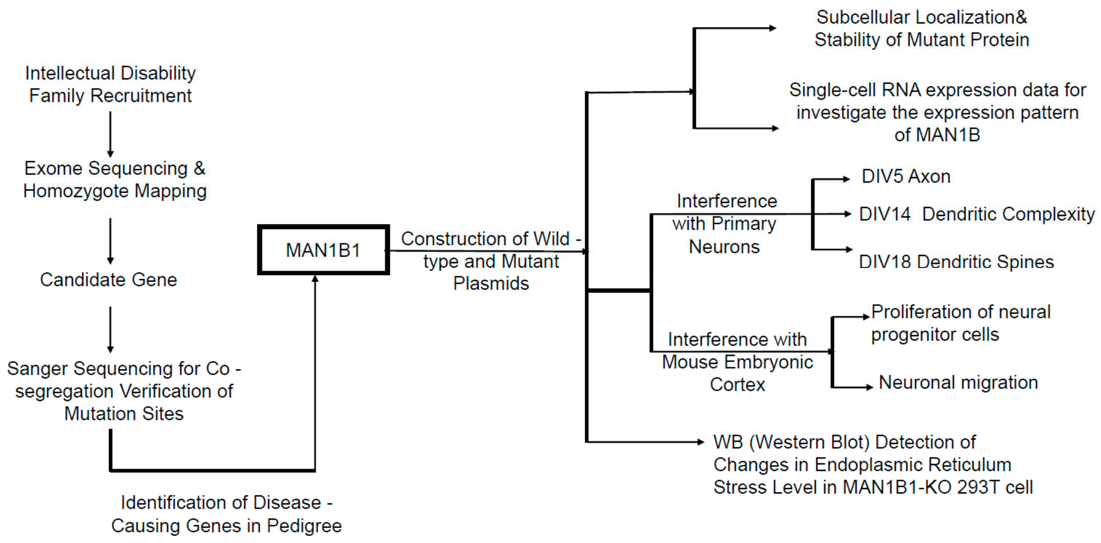

Supplementary Figure S4. Method summary flowchart.
